# Performance Analysis of Stop-Skipping Scheduling Plans in Rail Transit under Time-Dependent Demand

**DOI:** 10.3390/ijerph13070707

**Published:** 2016-07-13

**Authors:** Zhichao Cao, Zhenzhou Yuan, Silin Zhang

**Affiliations:** Ministry of Education (MOE) Key Laboratory for Urban Transportation Complex Systems Theory and Technology, Beijing Jiaotong University, Beijing 100044, China; zzyuan@bjtu.edu.cn (Z.Y.); 12114213@bjtu.edu.cn (S.Z.)

**Keywords:** stop-skipping, single line, scheduling, train path, tabu algorithm

## Abstract

Stop-skipping is a key method for alleviating congestion in rail transit, where schedules are sometimes difficult to implement. Several mechanisms have been proposed and analyzed in the literature, but very few performance comparisons are available. This study formulated train choice behavior estimation into the model considering passengers’ perception. If a passenger’s train path can be identified, this information would be useful for improving the stop-skipping schedule service. Multi-performance is a key characteristic of our proposed five stop-skipping schedules, but quantified analysis can be used to illustrate the different effects of well-known deterministic and stochastic forms. Problems in the novel category of forms were justified in the context of a single line rather than transit network. We analyzed four deterministic forms based on the well-known A/B stop-skipping operating strategy. A stochastic form was innovatively modeled as a binary integer programming problem. We present a performance analysis of our proposed model to demonstrate that stop-skipping can feasibly be used to improve the service of passengers and enhance the elasticity of train operations under demand variations along with an explicit parametric discussion.

## 1. Introduction

With the accelerated urbanization process, public transit as an important means of metropolitan environmental sustainability is increasingly of widespread concern. How to rationally carry out scheduling plans and how to estimate the performance comparisons of multiple forms has become a major public health topic area. Obtaining a reasonable scheduling plan is beneficial to public health in real-life since the performance analysis in needed, for example, to reduce environmental pollution, the energy consumption and generalized cost of public travelling.

High-speed rail (HSR) as mass public transit has been extensively developed over the last several decades and various available operation strategies have evolved. With the development of mass transit systems, a stop-skipping scheduling plan can greatly improve the efficiency and level of service (LOS). HSR scheduling plan is a principal stage, since its solution determines transit service quality and subsequent subproblems (i.e., the train and crew scheduling). Stop-skipping applied to HSR mainly focuses on station determination which aims at minimizing the total travel and waiting time experienced by passengers. This aspect has seen especially rapid development in Asia, where the passengers’ time and operators’ benefits are taken into account. It was first applied to the Chicago in 1947, but has since been abandoned in that system [1]. The definition of a stop-skipping scheduling plan can be comprehended using a simple expression: a decision will be made for scheduled trains to skip one or more stations if the total time can be reduced at the planning level. The total time in our further proposed model consists of total passenger waiting time and travel time for all scheduled trains. For one train, the skipped stations might serve significantly fewer passengers than those on the all-stops schedule, which might result in a shorter trip time for the train and a greater waiting time for the passengers at the skipped stations. For a scheduling plan, as a desired beneficial consequence, the LOS is improved for most of the users by the increase in the operating speed, which is the basis for enhancing the high density of train departures. In fact, the objective of stop-skipping scheduling plan is to achievea trade-off between adding the extra waiting time and reducing the travel time.

In high-speed systems, the rail-demand generally presents different shapes in space and time. This necessitates unique design approaches to provide users with a reasonable level of service. Compared with the standard operational plan (i.e., an all-stops schedule), the stop-skipping plan contributes to an unbalanced condition, in which the total travel time of passengers at congested stops is reduced since the relatively undersaturated stations are skipped, and their waiting times are eliminated. On the other hand, the development of the stop-skipping schedule offers a unique solution to high demand at peak hours, reaching a level at which the departure frequency and cruising speed cannot be further improved. To some extent, a stop-skipping schedule has greater applicability to the oversaturated demands of long-distance lines. We address the minimization of the total cost based on a given demand. Therefore, the plan’s hierarchical nature makes it possible to design stop-skipping schedules at a lower level and transit assignments at an upper level.

A stop-skipping scheduling plan consists of various modes, providing access to different trains to meet the travel demands for a given origin-destination pair. At the real-time operation level, the arrival of passengers at a station is not related to thearranged schedule because of noninterference, i.e., none of the individuals have information about the timetable for the trains before their arrival. However, the passenger behavior with regard to train or line selection is heavily affected by the stop-skipping type, platform structure, and arrival time of trains. Five stop-skipping forms are typically modeled for diachronic analysis.

Regarding the stop-skipping patterns for a one-way single track, the fundamental approaches are divided into deterministic and stochastic forms. The deterministic form is derived from the description and analysis given by Vuchic [2,3,4] in which stations along a line are classified into three groups, *A*, *B*, and *AB*. The trains in line *A* stop at the *A* and *AB* stations, while the trains belonging to line *B* stop at the *B* and *AB* stations. When they intend to alight at a B station, passengers boarding at an A station will transfer at an *AB* station onto line *B*. Thus, the need to walk to make this transfer might affect the attractiveness of stop-skipping. The main drawbacks of this form are the determination of the skipped stations and the potential to fall behind the estimated demand with respect to historical statistical data. In other words, the deterministic form is not able to perform rescheduling based on day-to-day or periodic demand assignments. Among the studies based on this form, readers can refer to Freyss [5], Lee [6], Suh [7], Vuchic [2,3,4], and Zheng [8]. In order to capture the characteristics of passenger flow fluctuation, a stop-skipping plan can be modeled using a stochastic form. When introducing the passenger dynamics, the skipped stations are specifically selected according to a stochastic model, which is likely better suited for demand assignments. Stochastic forms have been described using various mathematical models for more explicit exploration, even though the theoretical properties of this modeling approach are under investigation. Among the recent papers that use this approach, mention should be made of Chen et al. [9], Li et al. [10], Feng et al. [11], Liu et al. [12], Niu [13,14,15], Wang et al. [16], and Cao et al. [17]. A concise description and empirical quantitative comparison are presented below.

Five categories of the *AB* stop-skipping patterns are estimated with a continuous approximation approach for one-way single-track rail transit, with the goal of accelerating transit operations using fixed stopping schedules [5]. The five types of trips proposed by [18], which were based on different combinations of origins and destinations, were different from the five categories of stop-skipping operation strategies presented in our paper.

Constantin and Florian [18] formulated a mixed integer programming model for optimizing frequencies in order to minimize the total travel and waiting time of passengers; they proposed a projected sub-gradient algorithm for solving the problem. A robust timetable optimization model was proposed by Vromans et al. [19] involving the relationship between timetable reliability and timetable heterogeneity were examined via a number of simulation experiments. Mesa et al. [20] formulated a fleet frequency optimization model that integrates two control strategies, short-turning and expressing (or dynamic stop skipping), for the existing trip demand along a corridor. Their model was solved through a heuristic approach with three objectives: minimizing passenger overload, maximizing passenger mobility, and minimizing passenger loss. An optimization approach was proposed by Leiva et al. [21] for designing limited-stop services which minimized both the travel time of passengers and the operating cost of an urban bus corridor via optimizing the transport services of various types of buses with different arriving frequencies. Lin and Ku [22] proposed a stopping pattern optimization model seeking to maximize the profit made by a train company by determining the optimal train stopping strategy for a given OD (original-destination) demand. Then, they developed two genetic algorithms to solve the problem, namely binary-coded genetic algorithm and integer-coded genetic algorithm.

Wang and Lin [16] developed a bi-level model with the objective of minimizing the operating cost regarding the fleet size and total travel cost of passengers. Here the upper level of the model concentrated on determining service routes with the associated headways. The lower level referred to the route choice behavior, which was found by using Frank–Wolfe algorithm. Sogin et al. [23] proposed a mixed integer formulation to analyze an optimal balance between faster passenger travel times and lower service frequencies. The overall passenger travel times of the Metra Union Pacific North Line was decreased by 9.5% through a presented genetic algorithm.

Liu et al. [12] established a theoretical analytical context, in which an intermediate bus (among three successive buses) was able to use a stop-skipping strategy. Ingeniously, three objectives were optimized using a genetic algorithm incorporating a Monte Carlo simulation, including minimizing the total waiting time, in-vehicle travel time, and total operation cost. Sun et al. [11] proposed an optimization model for designing a skip-stop service that considered the bus capacity and overloads at bus stops. Then, only minimizing the total travel time for passengers was taken into account as the objective.

Parbo et al. [24] dealt with bus timetable bi-level optimization from the perspective of minimizing the waiting experienced by transfer passengers. The lower-level optimization was calculating transit assignments that yield the passengers’ route choice. A tabu search algorithm was adopted in order to avoid being trapped in local minima. Raveau et al. [25] modeled the route choice behavior in two metro networks (London Underground and Santiago Metro), by taking various attributes into consideration, including different time components, the transfer experience, the level of crowdedness, the network topology and other social-demographic characteristics. Anderson et al. [26] proposed a two-stage model accounting for the choice set generation and modeling route choice. The train choice behavior is estimated with regard to similarity of alternatives and heterogeneity of travelers using survey data. Clarifying the train choice behavior is crucial to understanding and predicting passenger preferences when they choose local/rapid/express trains for our proposed approach.

Using smart card-based automated fare collection data, Sun et al. [27] proposed three optimization models to design demand-driven timetables for a single-track metro service. The results showed that demand-dynamic timetables have great potential at reducing the waiting time of passengers and congestion on trains in Singapore’s mass rapid transit.

Chen et al. [9] aimed to optimize the headway of BRT along with a stop-skipping service based on the given OD demand matrices derived from morning peak hours. Binary variables representing whether or not a station is skipped by the stop-skipping service were introduced as inputs; the hypothesis is similar to that in our paper, but these binary variables are the output results of our proposed model. For the same stop-skipping service concept, Chen et al. [9] found the optimum headway to be between 3.5 and 5.5 min during the morning peak hour. However, our method clearly determines how to stop at or skip stations.

Niu [13] used a stop-skipping plan to solve a scheduling problem for a congested transit line and optimize the timetable. The fundamental mathematical mechanism was derived by [14]. In order to characterize the properties of passengers’ cumulative arrival variables, they innovatively defined the effective loading time slice by considered the bus capacity limit. However, the validity of expressing the passengers’ arrival form as an integral inequality remains to be proven; the objective of minimizing the overall waiting time was significantly less accurate and concise than the average waiting time [27] at approximately 50% of the headway. Furthermore, Niu et al. [15] focused on minimizing the total passenger waiting time at stations by computing and adjusting train timetables for a rail corridor by using given time-varying OD passenger demand matrices and skip-stop patterns.

Cao et al. [17] previously explored a mechanism for optimizing stop-skipping and proposed an approach to achieve equilibrium between avoiding stationary dwell time and producing additional waiting time. This equilibrium is likewise the core concept of the stochastic model. The model considers the acceleration and deceleration intervals. Utilization equilibrium was achieved for the performance of a schedule-based single-track in one direction. Given a simulation using a tabu search algorithm, two fundamental objectives were proposed for the stop-skipping schedule: minimizing the total waiting time of passengers, and travel time of passengers. An estimation based on empirical results showed that the stop-skipping scheduling plan is capable of optimizing the timetable. However, the problem of the study should be expanded by using an infinite constraint on the train’s capacity and extending the stop-skipping plan for a single train to all of the schemes or the entire network.

The issue is whether or not the advantage outweighs the inconvenience of transferring from line *A* to *B* or waiting for another train. In order to clarify the contradictory problem of whether a plan increases the operating speed, we considered scheduling plans for trains where different numbers of stations are skipped at different locations and determined the benefits.

Unlike existing literature where the stop-skipping scheduling service is optimized under only a sample given demand with the biggest impact on the planning results, we propose five optimized base forms in order to assess the potential effect from various time-dependent demand configurations. We found that the performance of the stop-skipping scheduling plan can be justified in many examples with mixed load patterns using deterministic and stochastic approaches.

**Figure 1:**
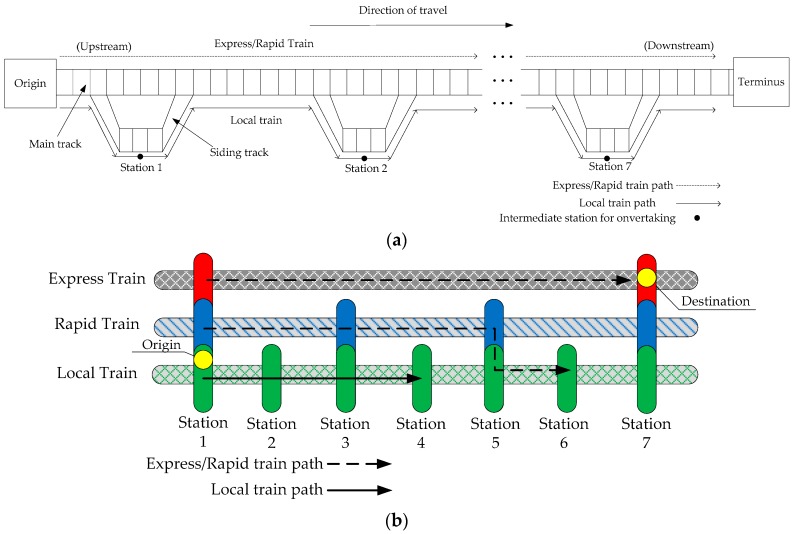
Stop-skipping represented by two fundamental graphs. (**a**) Topology of rail transit system investigated in this study; (**b**) Fundamental graph for five typical forms.

Figure 1a illustrates the topology of the railway system investigated in this study. Consider a situation in which trains are dispatched at regular intervals on a single, unidirectional track from an origin to a destination. Three types (categories) of trains are dispatchedand distinguished from stop-skipping services. No overtaking is allowed on the main track. However, all stations in the system are located on sidings, so it is possible for an express/rapid train to pass a local train at a station. Thus, a fast train’s passage of a station being served by a local train is unobstructed. Further, each station siding is sufficiently long so that braking and acceleration by a local train moving into or out of a station does not interfere with the operations of the express/rapid trains in the main track which the local train is leaving or entering.

Passenger’s train choice is identified in our model, which facilitates accurately the demand assignment to train paths for a single line even though without involving with the transit network route modeling. This behavioral information would be useful for improving the demand relationship management of the railway company and for optimizing train scheduling. Our approach can empirically capture passengers’ expected or experienced characteristics in the train path choice.

One major focus of this paper is comparing different approaches to optimizing the stop-skipping schedule at the planning level. One methodology based on the work of Vuchic [2,3,4] specifies that each train visits only a fixed subset of the stations, while another methodology described by Cao et al. [17] utilizes a stochastic selection methodology based on optimal evolution of the system conditions. The former represents a deterministic approach, and the latter represents a further developed stochastic one. Both approaches are embedded into a stop-skipping scheduling framework. This performance analysis allowed us to determine scenarios for which each of the two methods presented advantages to highlight their respective strengths for the construction of an integrated approach.

The stochastic method proposed by Cao et al. [17] is further developed, which has been complementally taken into account-congestion situation, train choice preferences and the train’s acceleration and deceleration process. The enhancement of the stochastic model might be an innovative contribution for the stop-skipping scheduling methodology. Next, our analysis for developing and performance assessment of efficient deterministic and stochastic forms for stop-skip scheduling plan turns out to be an innovative contribution provided our demonstrate convincingly its: (i) ability to reduce the risk of passenger overload on platforms and in the trains; (ii) feasibility and safety of the train path planning; and (iii) elasticity of train operations under demand variations. Moreover, demand assignment is the primary element for determining whether or how to adopt the stop-skipping scheduling plan. A significant finding is that the stop-skipping scheduling plan is more beneficial for a single line in which the demand distribution has distinct fluctuations.

As a sub-objective of our study, the stochastic model is further improved and better applied to the stop-skipping scheduling optimization, which is a beneficial supplement for stochastic approach. As a whole, the main objective of the paper is performance analysis of different proposed scheduling forms so as to find out the mechanism of determining whether or how to adopt the stop-skipping scheduling plan based on the diversified demand assignments.

## 2. Diachronic Cases and Assumption

We present several diachronic cases with seven stations and five trains to demonstrate the details of the performance by the five specific stop-skipping scheduling plans, based on the Taiwan’s rail transit system.

Based on the same given OD pairs, the benefits are estimated for the five forms of combinations, including both deterministic and stochastic ones. At the same time, five estimations are derived from one fundamental graph, as shown in Figure 1b.

The performance analysis is carried out empirically using the empirical study of Taiwan railways. As noted by Asakura et al. [28] and Kusakabe et al. [29], some railways in metropolitan areas have several different types of trains, designated as express, rapid, and local trains. Figure 1b shows that these rail trains utilize three modes, with local trains stopping at all of the stations, and rapid and express trains passing some stations to achieve shorter travel times than the local trains. The express trains stop no intermediate stations. Moreover, the local train mode is generally called the standard operation or all-stops plan. The stop-skipping schedule is determined in advance and not in real-time during the planning level. The approach to the scheduling plan greatly depends on historical passenger data, such as that gathered from smart card and automated fare collection (AFC) systems. Transit operators provide the stop-skipping information to passengers via panels at platforms and announcements in the trains. The drivers and crew have full knowledge of the train schedule because of the predetermined timetable.

**Figure 2:**
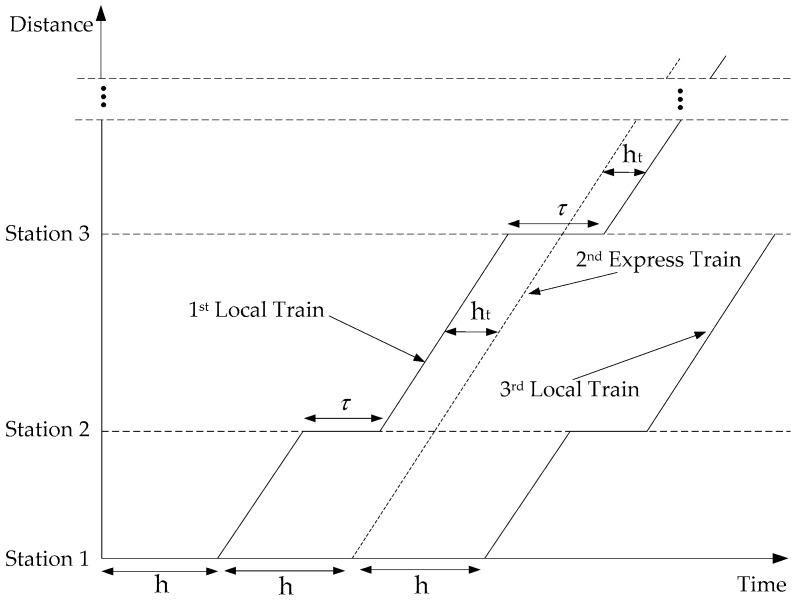
Minimum headway $h_{t}$ depicted as a time-space diagram.

Based on the pedestrian transportation dynamics [30], we employed the following assumptions to avoid choice-confusion due to the stop-skipping schedule:

(I) All passengers desire to minimize both their waiting and travel times on the train, which should be accommodated within a certain extent, such as 10 min.
(II) The timetable can be illustrated as a parallel train working graph. In other words, the average operating speed is consistent between adjacent stations, as shown as Figure 2.
(III) The train dwell time is sufficient for all of the waiting passengers to board. Our study showed that there is no point in adding extra waiting time for passengers who are skipped by previous rapid or express trains.
(IV) The trains have an infinite capacity to load the waiting passengers. Infinite capacity is unrealistic, but in theoretical context, this assumption can substantially reduce the complexity of the model. Moreover, the passengers could board the trains as long as they take the tickets with ignoring the effect of overloading.
(V) No overtaking is allowed on the main track while all stations in the system are located on siding tracks to enable overtaking of trains. Further, each station siding is sufficiently long so that braking and acceleration by a local train moving into or out of a station does not interfere with the operations of the express/rapid trains.
(VI) The configurations of different demand assignments are given with accordingly determined train choices.
(VII) Passengers have no preference regarding train classes. When a passenger arrives at a station, he or she will board the first train available at the station.

The case is derived from the empirical statistics of a real sectional rail line in Taiwan, China. In order to find an optimal operation strategy, we compare the overall benefits of five mainstream forms of fundamental operations, including all-stops and stop-skipping forms. These are given below:

1st form: Only local trains run on the rail line.
2nd form: Both local and rapid trains run.
3rd form: Local and express trains operate alternately.
4th form: Local trains, a rapid train, and an express train run.
5th form: Based on passenger demand, the skipped stations are selected according to a stochastic approach.

As shown in Figure 1b, there are three types of trains. An express train only stops at the origin and terminal stations (i.e., the 1st and 7th stations). A rapid train only stops at every second station (i.e., the 1st, 3rd, 5th and 7th). A local train stops at all stations. We propose an applicable model and algorithm for 5th form to show its complexity.

## 3. Sketch of Mathematical Model for 5th Form

The variables are listed in Table 1.

Our proposed approach to describe the stop-skipping problem is introduced in detail as follows. The total dwell time of trains is derived from the number of skipped stations, which is expressed in Equation (1):

(1) $$\tau_{i,j}=\;y_{i,j}\cdot \tau ,\;i=1,2,\ldots ,M;\;j=2,3,\ldots ,N$$

Equations (2) and (3) act as constraints to track the departure time $D_{i,j}$ and arrival time $A_{i,j}$ of train *i* at station *j*, respectively:

(2) $$D_{i,j}=\;A_{i,j}+\;\tau_{i,j}$$

(3) $$A_{i,j}=\;D_{i,j-1}\;+\;r_{j}\;+\;\frac{v_{\max}}{432}\cdot \frac{1}{\beta }\cdot y_{i,j}\;+\;\frac{v_{\max}}{432}\cdot \frac{1}{\partial }\cdot y_{i,j-1}$$

Here, $D_{i,j}$ is the departure time of train *i* from the previous stop *j*, and *r_j_* is the travel time between the two stations, $y_{i,j-1}\cdot v_{\max}/(432\cdot \partial )$ is the acceleration time at station *j* – 1 and $y_{i,j}\cdot v_{\max}/(432\cdot \beta )$ is the deceleration time at station *j*. Without loss of generality, $\frac{v_{\max}}{432}\cdot \frac{1}{\beta }\cdot y_{i,j}+\frac{v_{\max}}{432}\cdot \frac{1}{\partial }\cdot y_{i,j-1}$ (min) is the inevitable time loss consisting of both braking and acceleration losses as a result of stopping at one station. To fulfill the tracking requirements, the arrival time is calculated using Equation (3). The parameter “432” need to be explained based on Vuchic [4]. It is an important variable of transferring the start-up and braking process into the time of acceleration and deceleration.

The total number of passengers skipped by train *i* at station *j* is:

(4) $$S_{i,j}=\sum_{K=j+1}^{N}S_{i,jk}$$

The number of passengers boarding train *i* at station *j* is:

(5) $$B_{i,j}=y_{i,j}\sum_{K=j+1}^{N}W_{i,jk}(\xi +\eta \cdot y_{i,k}),\;i=1,2,\ldots ,M;\;j=1,2,\ldots ,N-1$$

The number of passengers picked up by train *i* at station *j* is:

(6) $$V_{i,j}=y_{i,j}\sum_{K=1}^{j-1}W_{i,kj}y_{i,k},\;i=1,2,\ldots ,M;\;j=1,2,\ldots ,N$$

Then, the number of passengers skipped by train *i* at station *j*, who expect to alight at station *k* is:

(7) $$S_{i,jk}=W_{i,jk}-W_{i,jk}\cdot y_{i,j}\cdot (\xi +\eta \cdot y_{i,k})$$

The studies of Liu et al. [12] and Niu et al. [13] remarked on the absoluteness of the number of boarding passengers. In other words, passengers do not board a train that skips the station where they want to alight, i.e., $S_{i,jk}=W_{i,jk}-W_{i,jk}\cdot y_{i,j}\cdot y_{i,k}$. If stations *k* and *j* are not skipped simultaneously, $S_{i,jk}=0$, while $S_{i,jk}=W_{i,jk}$ explains that only station *k* or *j* is skipped. However, these train choice behaviors might be inappropriate because the passengers’ perception is neglected. Determining the excepted or experienced user perception while accounting for seamless travel via all-stops trains or alternative time-saved travel via transfers is a trivial challenge. The two prediction parameters ξ and η are proposed to reflect to generate choice sets in order to estimate passengers’ perception with regard to choosing the current train or the next one. If all passengers prefer to take relatively fast trains without interest in the all-stops trains, the parameters ξ and η are set to 0% and 100%, respectively. This hypothesis was adopted by Liu et al. [12] and Niu et al. [13], but this impractically neglects passengers’ perception. The current study challenged the previous hypothesis by generating choice sets using perceived cost described as Equations (8)–(10).

The perceived cost *C_i_* of choosing the current train *i* is equal to the weighted average of the experienced cost *e_i_* and the expected cost *E_i_*. The parameter π represents the weight of the experienced actual cost in updating the perceived cost. A large π describes the behavior with a strong habitual tendency, while a small π means that passengers depend more on recent expectation (or the previous experienced cost is too high):

(8) $$C_{i}=\pi e_{i}-(1-\pi )E_{i}$$

The probability ξ of choosing the current train *i* by the waiting passengers can be calculated with the following equation:

(9) $$\xi =\frac{e^{C_{i}}}{e^{C_{i}}+e^{C_{i+1}}}$$

The logit model is applied, leading to the proportion of demand ξ. Then the probability η of choosing the next train *i* is:

(10) η=1−ξ

In addition, *S_i,jk_* = 0 if neither station *k* or *j* is skipped, as given in Equation (7). If the departing station *j* is skipped, then *S_i,jk_* = *W_i,jk_*. In particular, if *k* is similarly skipped, the result (*S_i,jk_* = *W_i,jk_*) might lead to a impractical train choice behavior. This means that some passengers do not board train *i* because their origin station is skipped. Then, if their destination station is skipped by train *i*+1, the passengers would again not board this train. However, this is unrealistic based on Assumption (I). In fact, approximately 50% of the passengers might board the train, even if their destination station is skipped. They will alight at the next or previous station, or be served by another feeder service. Here, *W_i,jk_* is the total number of passengers waiting at station *j* and expecting to alight at station *k*:

(11) $$W_{i,jk}=\;S_{i-1,jk}+\lambda_{j,k}h,\;i=1,2,\ldots ,M;\;j=1,2,\ldots ,N;\;k=2,3,\ldots ,N$$

When some stations are skipped by a train, passengers expecting to board at those stations just have to wait for at least one more headway to be served. The stop-skipping schedule problem involves determining the skipped stations and planning schemes. Evidently, there is an optimal equilibrium between reducing the trip time by stop-skipping and increasing the waiting time compared with the all-stations scheme. Li et al. [30] proposed the idea that adding one stop means that one train has to be dropped from a given train-diagram for a high-speed railway. We propose an integral formulation for the optimization problem and developed algorithm.

## 4. Objective Function

For the formulation, we define $\lambda_{j,k}$ as the passenger boarding rate from station *j* heading to *k*. Conventionally, the average waiting time is set to ${{\bar{w}}_{i}}_{j}=h/2$ at station *j* waiting for train *i*. For train *i*, when station *j* is skipped, *S_i,j_* passengers have to wait for *h*/2 + *h* headways. Nevertheless, the rest of the passengers wait for half of a headway (*h*/2) without skipping. Thus, the total waiting time for passengers taking trains *i* and *i*+1 is:

(12) $$Z_{1}=\sum_{i=1}^{M}\sum_{j=1}^{N}\left[(B_{i,j}-S_{i-1,j})\frac{h}{2}+S_{i-1,j}\cdot \frac{3h}{2}\right]$$

The total trip time for passengers boarding train *i* at station *j* and alighting at station *k* is $\sum_{f=j+1}^{k}(r_{f}+\frac{v_{\max}}{432}\cdot \frac{1}{\beta }\cdot y_{i,f}+\tau_{i,j}\cdot y_{i,f}+\frac{v_{\max}}{432}\cdot \frac{1}{\partial }\cdot y_{i,f-1})$. By summing all of the trains, the travel time for all of the passengers is calculated as follows:

(13) $$Z_{2}=\sum_{i=1}^{M}\sum_{j=1}^{N-1}\sum_{k=j+1}^{N}W_{i,jk}\cdot \sum_{f=j+1}^{k}(r_{f}+\frac{v_{\max}}{432}\cdot \frac{1}{\beta }\cdot y_{i,f}+\tau_{i,j}\cdot y_{i,f}+\frac{v_{\max}}{432}\cdot \frac{1}{\partial }\cdot y_{i,f-1})$$

These two objectives are addressed using the weighted sum method:

(14) $$\begin{array}{l} \min Z=c_{1}Z_{1}+c_{2}Z_{2}=c_{1}\sum_{i=1}^{M}\sum_{j=1}^{N}\left[(B_{i,j}-S_{i-1,j})\frac{h}{2}+S_{i-1,j}\cdot \frac{3h}{2}\right]\\ +c_{2}\sum_{i=1}^{M}\sum_{j=1}^{N-1}\sum_{k=j+1}^{N}W_{i,jk}\cdot \sum_{f=j+1}^{k}(r_{f}+\frac{v_{\max}}{432}\cdot \frac{1}{\beta }\cdot y_{i,f}+\tau_{i,j}\cdot y_{i,f}+\frac{v_{\max}}{432}\cdot \frac{1}{\partial }\cdot y_{i,f-1}) \end{array}$$

where *c*_1_ and *c*_2_ are the weighting values of time for each objective. Furthermore, the first and last stations cannot be skipped, thus:

(15) $$y_{i,1}=\;1,\;y_{i,N}=\;1$$

Moreover, two consecutive trains do not skip the same station, so:

(16) $$y_{i,j}+\;y_{i+1,j}\geq 1,\;i\;=\;1,2,3,...,N-1$$

Considering the feasibility of the stochastic form, the constraint that two successive stations cannot be skipped is expressed as follows:

(17) $$y_{i,j}+\;y_{i,j+1}\geq 1,\;j=1,2,3,...,N-1$$

Figure 2 presents a time-space diagram of the minimum headway depicted later. Each local (rapid/express) train is denoted using a solid (dashed) line. The slopes of all lines between stations are equal because all trains move at the same speed on the main line. The minimum headway $h_{t}$ is the minimum time interval between two successive trains so that they can enter and depart from a station safely. Due to assumption (V), a train cannot enter the main track until a minimum headway after the preceding train’s departure. With the stop-skipping plan, the minimum headway between two consecutive trains is in fact affected by whether a train stops at or skips a station. The last two constraints Equations (18) and (19) are disjunctive constraints that enforce the headway restrictions on the main line between origin and terminal station. In particular, these constraints guarantee that (express/rapid) train *i* enters main track either $h_{t}$ minutes after (constraint Equation (18)) or $h_{t}$ minutes before (constraint Equation (19)) a preceding train. The minimum headway constraint can then be described as:

(18) $$(i-1)h-\sum_{k=2}^{j}\tau_{k}+Mx_{i,j}\geq h_{t}$$

(19) $$(i-1)h-\sum_{k=2}^{j}\tau_{k}-M(1-x_{i,j})\leq -h_{t}$$

Obtaining a fast solution algorithm is beneficial to solving our proposed mixed nonlinear binary programming problems. These are difficult to solve with an exact algorithm because the objective functions are not convex or concave. Hence, we present a tabu search algorithm to solve our model in the next section.

## 5. Algorithm for the Stochastic Model

**Figure 3:**
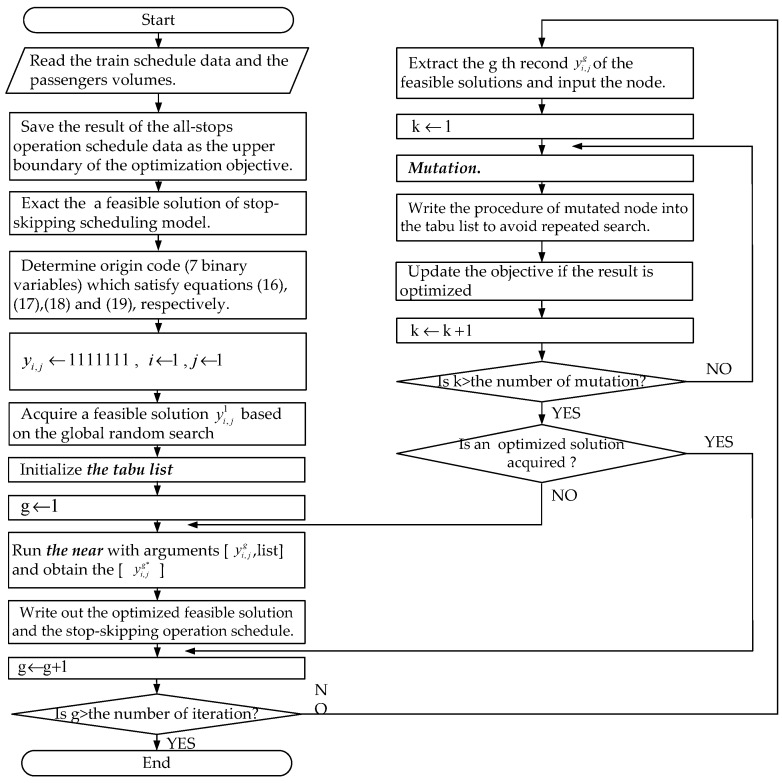
Flowchart of the tabu algorithm.

Our proposed model is a nonlinear programming problem with two mixed-binary objective functions and closely related parameters. It is difficult to solve using conventional solution approaches. A tabu algorithm, which generates binary solutions with an ergodic search, contributes to the stop-skipping problems. With no loss of generality, the algorithm can solve problems of forms 1st–4th by adopting a deterministic 0/1 selection set. The main steps of our proposed algorithm are described in the form of a flow chart in Figure 3. The details are presented in the Appendix (Table A1, Table A2, Table A3 and Table A4).

*Step 0:*  (Initialization). Check whether or not the problem is feasible (constraints Equations (1)–(19)), and input the data structures, including the number of iterations, mutations, and neighborhood-solution comparisons. The initial solution is randomly generated from the input matrix. The tabu list is set to zero, and the iteration number *g* = 0.
*Step 1:*  (Obtaining the objective constraint). The total all-stops operation time is calculated as the maximum of the objective function. Then, *g = g* + 1, and the test begins.
*Step 2:*  (Seeking a feasible solution). A global search is iterated. A feasible solution is found and recorded as one of a number of solutions.
*Step 3:*  (Optimizing the feasible solution). The feasible solution should be optimized by comparing neighborhood-solutions under repeatedmutation. Either the feasible solution is optimized to obtain a better consequence or to avoid deviating too much from the existing solution, only two elements of the outcome matrix are changed to their alternate value (0 or 1).
*Step 4:*  (Stopping the test). When the minimum objective function *Z* cannot be further reduced using the above steps or the number of iterations reaches the predefined upper bound, the matrix *Y_ij_* can be considered to be one of the optimal solutions. Finally, the stop-skipping problem is solved by employing the tabu search algorithm.

## 6. Computational Results for Cases

We present a real-life case study whose data refer to Chang et al. [31] to explain how the model works. To evaluate five forms and the feasibility of our proposed algorithm, we used a one-way single-track rail for the performance analysis [32].

Normally, the desired schedule is related to the design frequency [33], which directly depends on the trip time of a train during an operation period. Therefore, opportunities theoretically exist to dispatch more trains in stop-skipping forms. By using an intuitively horizontal comparison, the analytical expressions for five trains associated with these five forms were summarized.

For the 7-station case, Table 2 and Table 3 lists the relative parameters including inputs of the model, distance (km), train running time (min), and planned hourly passenger volume (passenger-trip/h) between two stations respectively. In order to facilitate problem formulation and simplification, this study assumes that the passenger arrival pattern at stations follows some uniform probability distributions.

**Figure 4:**
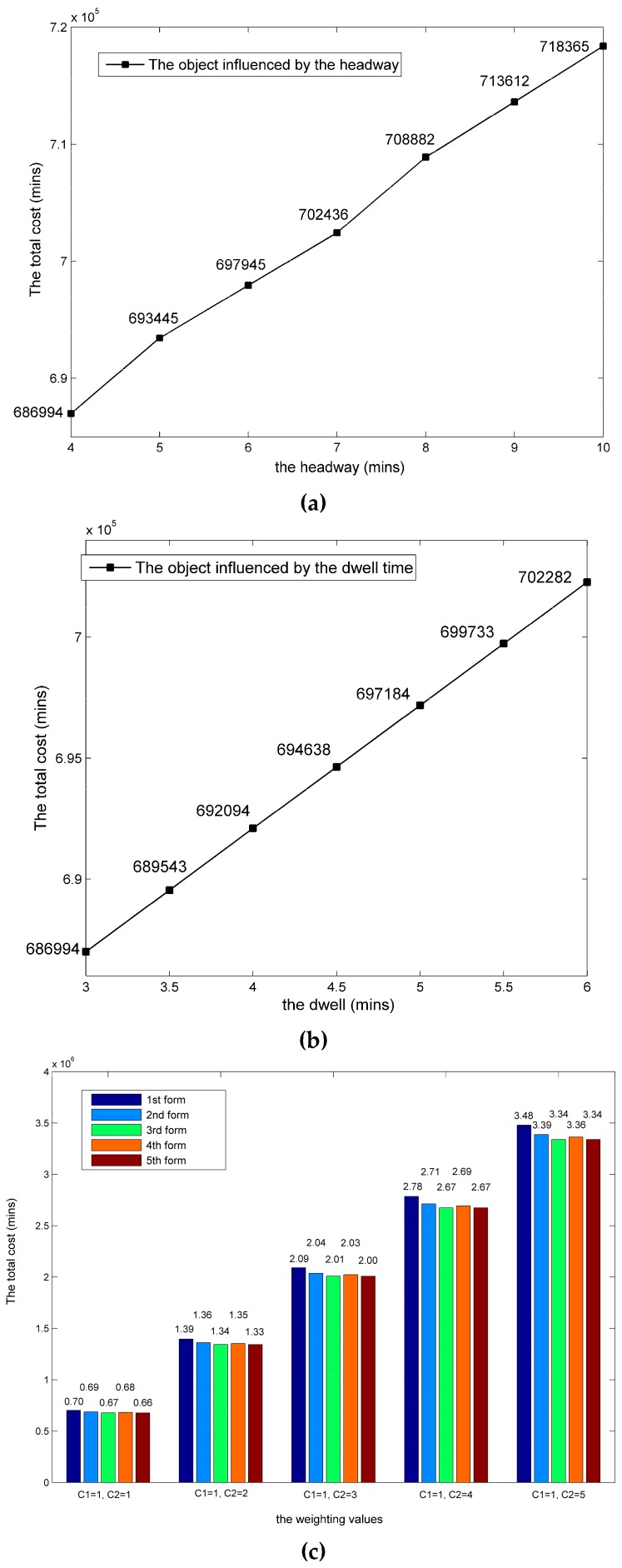
Objective versus multiple parameters changing. (**a**) Objective versus dwell time; (**b**) Objective versus headway; (**c**) Objective versus weighting values (*c*_1_,*c*_2_).

Weighted values *c*_1_ and *c*_2_ express the value of time (VOT) under different circumstances [26]. A substantial amount of research [2,3,4,21] states that a value of 2 is commonly used as a factor to calibrate the waiting time *c*_1_. This means that passengers rate 1 min of waiting as equivalent to 2 min of driving. Accordingly, the two weighting values in Equation (14) are set as *c*_1_ = 1 and *c*_2_ = 2. To compare the performance of the five forms of stop-skipping scheduling, the results of the multi-plans are listed in Table 4 and Table 5. Our presented approach corresponds to 5th form with distinct variations. No improvement could be achieved using our proposed algorithm after 146 iterations over about 4 min on a personal computer with an Intel (R) Pentium (R) CPU running at 2.90 GHz with 4.00 GB of RAM. Moreover, the convergence effect of the first train in 5th form was achieved by using our algorithm as a representative illustration, as shown in Figure 4. The results showed good convergence of the iterative process, and the best values for all of the generations are listed below.

In order to make good use of the tabu algorithm, tests needed to be run to calibrate some appropriate parameters. The tabu algorithm was run with *g* = 50, 100, 200, 500, and 1000 iterations. The results showed that the most appropriate iteration number was 200. Then, the tabu algorithm was run with mutation population sizes of 100, 300, 500, and 1000 generations. The results demonstrated that a population size of 300 resulted in a lower processing time and greater efficiency.

Statistical data from the simulation results are listed in Table 4 and Table 5. The initial term quantifies the total passenger waiting time, which depends on the number of skipped stations, and 1st form experiences the maximum duration. 2nd term measures the aggregate passenger travel time, and 5th form is the optimal choice overall as a result of the flexible stop-skipping strategy. Three different train paths were tested: (i) the local train had the shortest waiting time and longest travel time for passengers; (ii) the rapid train increased the total delay (extra waiting time) by approximately 104% while saving 6.9% of the travel time; and (iii) the total waiting time was drastically increased by 129% with the express train, which increased the total travel time by 11%. Note that the traveling time increaseses more than the waiting time (almost 2%) for all three train paths.

The results showed that 5th form optimizes the objective function. Intuitively, 5th form can save 3.26% compared with 1st form for the case with five trains. This is an important contribution to optimizing the stop-skipping problem but is achieved at the expense of a plan scheduled according to the rule of time-dependent varying demand.

Regarding the resulting time, if one schedule determines a stop or skip action at a certain station that is not physically appropriate, then a stop-skipping schedule will not contribute much in relation to saving time. In other words, the total time of passengers in 2nd, 3rd, and 4th forms save 1.76%, 2.09% and 2.23% than 1st form. The conflict interest space is so limited that the total travel time of stop-skipping schedules could not be sharply reduced in large magnitude. In an attempt to make trade-offs between these two conflicting objectives, the best-compromise solution is the stochastic form. In 5th form, the 1st and 2nd trains depart alternately as a cyclic schedule, which might be unobstructed at adding operation’s complexity.

For 3995 pax in one form of five trains, we observe some conclusions to prove more efficient performance of stop-skipping train service in the following indicators: average trip distance, waiting/travel time per passenger and transport occupancy level per train. Independently of the average trip distance to be identified, the results demonstrate that a rapid/express train facilitates a longer distance trip (561.2/617.1 km per passenger) than a local train (490.3 km per passenger). While boarding a rapid/express train might result in sacrificing a little extra waiting time for a passenger (2.0/2.6 min per passenger), the fast service achieves great benefit for average travel time yielded lower than a local train (12.3/19.7 min per passenger). Moreover, a more balanced performance in the stochastic form is even noticeable. With a passenger only experiencing extra waiting time (0.5 min), a significant reduction of travel time (7.6 min) would be improved. Fluctuations of occupancy level per train can be justified from alternant dispatches between a rapid/express train and a local train. The 2ndform shows the benefits of attenuating the loading unbalance.

## 7. Discussion of Different Constraints

A mathematical model with a wide range of applicability is provided to solve stop-skipping problems. In practice, two or more stations of the Taiwan Rail Transit system might be skipped consecutively. Table 4 and Table 5 list the results for the stochastic form when two or three stations are skipped successively. The total time of passengers *Z*^2^ for one train trip is equivalent to 132,419 min (Table 6(a)), and *Z*^3^ = 131,812 min (Table 6(b)).

The objective function can be further optimized through these numerical simulations, because the given constraints are relaxed, i.e., $y_{i,j}+y_{i,j+1}+y_{i,j+2}\geq 1,\;j=1,2,3,...,N-2$ (Table 6(a)) and $y_{i,j}+\;y_{i,j+1}+\;y_{i,j+2}+\;y_{i,j+3}\geq 1,\;j=1,2,3,...,N-3$ (Table 6(b)). If stations can be skipped successively in the uncontrolled case, all intermediate stations are skipped with the minimum value 123,579 min, which is also express train service.

## 8. Evaluation of Other Influences

The dwell time and headway significantly affect the objective [34]. Therefore, we need to analyze these two parameters in relation to the sensitivity of the stop-skipping model. To establish the trend of the function, we acquire a series of values to observe the changes in the total cost, which is shown in Figure 4. The objective is positively related to these two parameters, namely the dwell time and headway. Furthermore, the objective can vary by 0.7420% and 0.6919% on average when the dwell time and headway change by 1 min, respectively. Therefore, the most sensitive parameter, the dwell time, affects the objective of our model, which is important to the Operations Management Department. Moreover, the stop-skipping schedule is depreciated by a long headway, because the waiting time is too long to make it attractive.

A statement is added addressing how the objectives vary if the multiple (*c*_2_) is different, such as, 1.0, 2.0, 3.0, 4.0 and 5.0, shown as Figure 4c. The 3rd form shows its superiority to others which indicates the long-distance passengers concerning about their travel time is benefit from the express trains skipping more stations. Furthermore, with the multiple (*c*_2_) increasing, the cost of 1st form rises dramatically for which the standard (all-stops) solution delays more travel time due to more stops. Certainly, the multiple (*c*_2_) should be set depending on the application scenarios. Urban rail travelers may be more sensitive to waiting time but the headway is shorter so there is an expectation that the traveler can just arrive at the station and wait for the next train. Then, the value (*c*_2_) could be reduced as a rationally boundary from 1.5 to 2.0 unit. In the high speed rail, the value is set as 2.0 unit and more, which represents the emphasis of long travel time.

**Figure 5:**
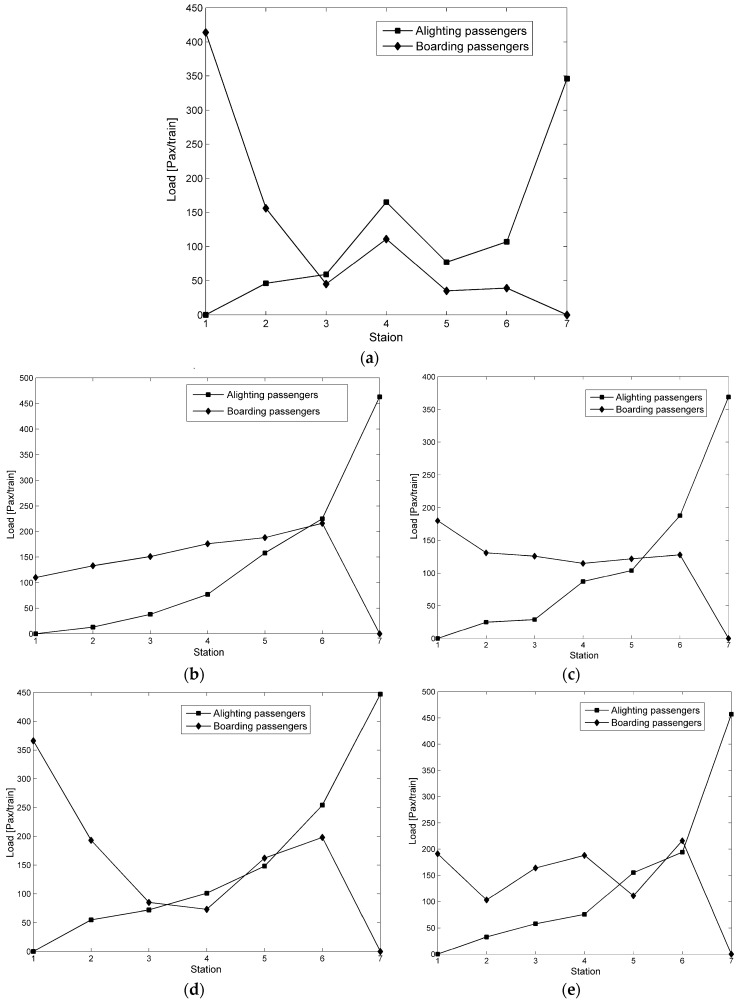
Load profiles of time-dependent demands. (**a**) Example 1; (**b**) Example 2; (**c**) Example 3; (**d**) Example 4; (**e**) Example 5.

The methodology developed in our study is applied to the aforementioned rail line comprising sevenstations, with four other different time-dependent demand scenarios that reproduce the concentration patterns of various stop-skipping plans, which are typical of real scheduling performance. The demand profiles are shown in Figure 5, where Example 1 is a real case (corresponding to the Taiwan Rail Transit), which was already discussed in the previous section, while the other examples are hypothetical. In terms of the spatial configurations of different demand assignments, the shape of the accumulation of boarding passengers is given more attention than that of alighting passengers, which is a method to distinguish the five examples. Example 1 is a radial corridor toward the suburbs during the morning peak hour. Examples 2 and 3 show homogeneous demand assignments at the peak and non-peak times. Examples 4 and 5 show the typical peak-demand route patterns. The performance of the five stop-skipping scheduling plans is later numerically verified under different time-dependent demands.

Integrating the results of Examples 1–5 shows that the optimal stop-skipping patterns, i.e., 2nd–5th forms, are superior to the all-stops schedule, out of which the stochastic form may be the best candidate for passengers and operators. Moreover, 3th form is the best of the deterministic plans. Table 7 lists the distinct results obtained from Examples 2 and 3 for different demands. The stop-skipping plan will be more attractive during a high-demand period. The total time can be reduced by 2%–5% in Example 2, compared with a 1% reduction in the total savings for Example 3. Thus, the stop-skipping plans produce schedules with high frequencies and short headways, leading to the alleviation of congestion during the peak hours. Examples 4 and 5 were generated from the peak-demand patterns.

There is little difference between the single-peak (Example 4) and bimodal (Example 5) demand configurations because the average passenger costs (138.1–146.6 min/passenger) would be nearly equal. If two or more stations are skipped consecutively, even deadheading, the performance is further optimized for unbalanced demand scenarios. We can obtain conclusions about the demand conditions with regard to which stochastic stop-skipping schedule is best for balanced and high-demand conditions. In all cases, 5th form performs better than the others for all five examples. The analysis of the pre-planned assignments shows that the stop-skipping scheduling plan can deal with intractable demand imbalances in sections and periods.

## 9. Conclusions and Future Research

In this paper, we addressed the problem of formulating a stop-skipping scheduling plan for a rail transit system that uses various forms and scenarios to minimize the total travel time of the passengers. There are two main contributions on the overall stop-skipping scheduling methodology. On one hand, the empirical operational technique is described more sophisticated in our developed model. On the other hand, our study could help operators to execute which stop-skipping scheduling plan as an optimal solution based on what is the demand distribution during the planning level.

While this scheduling concept has been successfully used in previous transit plan studies, the new proposed model explicitly captures the stochastic nature of the stop-skipping strategy. No such analytical method has previously been developed to differentiate the stochastic and deterministic forms. The absence of a stochastic stop-skipping system in a rail transit network tends to result ina stereotypical plan because it fails to capture the nature of the demand [11,13,14,15]. Conventional approach is acquired based on the static demand assignment, which hardly handles the dynamic flow regularity. An amendment of its drawbacks is proposed by our model.

The stochastic method proposed by Cao et al. [4] is further developed, which has been complementally taken into account-congestion situation, train choice preferences and the train’s acceleration and deceleration process. The enhancement of the stochastic model might be an indispensable improvement for the overall stop-skipping scheduling methodology. In particular, our analysis for developing and performance assessment of efficient deterministic and stochastic forms for stop-skip scheduling plan turns out to be an innovative contribution provided our demonstrate convincingly its (i) ability to reduce the risk of passenger overload on platforms and in the trains; (ii) feasibility and safety of the train path planning; and (iii) elasticity of train operations under demand variations. Moreover, the passenger characteristics are described based on the OD demands for passengers. Demand assignment is the primary element for determining whether or how to adopt the stop-skipping scheduling plan. A significant finding is that the stop-skipping scheduling plan is more beneficial for a HSR line in which the demand distribution has distinct fluctuations.

It was found that the core problem of our study was whether the extra waiting time produced by the stop-skipping scheduling plan could be compensated for by the time saved when traveling. The total trip time was considered to be the estimated service level of the rail transit. Interestingly, the OD demand was found to have a large influence on the stop-skipping scheduling plan. In other words, skipped stations showed diverse performance during the peak hours, non-peak hours, and weekend hours, during which the passenger flows fluctuated. Therefore, five forms of the proposed stop-skipping scheduling plans were compared and quantitatively analyzed using five examples. The performance of the deterministic forms was less favorable for stop-skipping operations, even though passengers could easily understand and adapt to them. On the other hand, the stochastic form had a better performance than the deterministic forms and standard (all-stops) operation.

An optimized function with two objectives was developed to find the optimal stop-skipping plan under empirical demands. The passenger travel and waiting times and the trains’ trip time were estimated. A tabu algorithm was used to solve the optimization model. A numerical example was used to confirm that the tabu algorithm could obtain a satisfactory performance with more efficiency. Compared to the deterministic forms and all-stops operations, the stochastic form showed the best results with the minimum travel times. We accurately calculated a viable stop-stopping strategy and demonstrated the benefits of the stochastic form because the stop-skipping operation is a very complex problem and is related to demand assignment.

Our stop-skipping model has not previously been applied to a real-life transit network in Asia because it would aggravate the operation complexity. Future research will include extending our approach to different marshalling plans, considering the available capacity, and developing efficient solution algorithms for large-scale implementation.

## Figures and Tables

**Table 1:** Presentation of variables.

Variables	Explanations
*j*	Station number of line *i, j = 1, 2, …, N*
$\tau_{i,j}$	Dwell time of train *i* at stop *j*; uniform dwell time is *τ*
*V_max_*	Maximum speed of the trains, km/h
$r_{j}$	Travel time between stations *j−1* and *j, j = 2,3,…,N*
$S_{i,jk}$	Number of passengers skipped by train *i* at station *j*, who intend to alight at station *k*, *1 ≤ j < k ≤ N*
$S_{i,j}$	Total number of passengers skipped by train *i* at station *j*
$B_{i,j}$	Number of passengers boarding train *i* at station *j*
$W_{i,jk}$	Number of passengers on train *i*, boarding at stop *j* and about to alight at station *k*, *1 ≤ j < k ≤ N*
$\lambda_{j,k}$	Arrival rate of passengers heading to station *k* from station *j*
ξ,η	Weight factors depending on the situation, in which the destination station is skipped
$y_{i,j}$	A binary variable for the stop-skipping decision of train *i* at stop *j*; i.e., *y_i,j_* = 0 if stop *j* is skipped and *y_i,j_* = 1 otherwise
*M*	A large positive number
$x_{i,j}$	If (express/rapid) train *i* arrives at intermediate station before a (local) preceding train departs from this station *j*, $x_{i,j}=1$ and otherwise (binary) $x_{i,j}=0$
$h_{t}$	Minimum headway in minutes between trains on main track (real, >0)

**Table 2:** Input parameters of case study.

Parameter	Value	Unit
Set of stations (*N*)	{1,2,3,4,5,6,7}	-
Operating hours, h	1	h
Train capacity	900	seats/train
Frequency and service line	15	trains/h
Dwell time (τ)	3	min
Headway (*h*)	4	min
Minimum headway ($h_{t}$)	2	min
Weighting values (*c*_1_)	1	*-*
Weighting values (*c*_2_)	2	*-*
Acceleration (∂)	0.9	m/s^2^
Deceleration (β)	1.0	m/s^2^
Maximum speed of the trains (*V_max_*)	240	km/h

**Table 3:** Distance, train running time, and planned hourly passenger volume between stations (*N* = 7).

Adjacent Stations	1	2	3	4	5	6	7
1	(0,0,0)	(35.8,14,683)	(65.6,27,737)	(159.5,40,1407)	(245.1,65,483)	(307.4,83,636)	(338.1,84,2257)
2	(35.8,14,697)	(0,0,0)	(29.8,11,149)	(123.7,36,748)	(209.3,61,271)	(271.6,79,305)	(302.3,83,861)
3	(65.6,27,603)	(29.8,11,111)	(0,0,0)	(93.9,24,320)	(179.5,48,53)	(241.8,67,64)	(272.5,71,242)
4	(159.5,40,1298)	(123.7,36,731)	(93.9,24,337)	(0,0,0)	(85.6,22,345)	(147.9,40,413)	(178.6,44,900)
5	(245.1,65,340)	(209.3,61,189)	(179.5,48,45)	(85.6,22,246)	(0,0,0)	(30.7,11,817)	(93.0,29,332)
6	(307.4,83,513)	(271.6,79,270)	(241.8,67,68)	(147.9,40,295)	(62.3,17,222)	(0,0,0)	(30.7,11,591)
7	(338.1,84,2105)	(302.3,83,776)	(272.5,71,241)	(178.6,44,768)	(93.0,29,465)	(30.7,11,817)	(0,0,0)

**Table 4:** Results of five forms (unit: min).

Forms’ Categories		List	Types of Trains	Average Trip Distance	Waiting Time Per Passenger	Travel Time Per Passenger	Occupancy Level	Total Passenger Waiting Time (Z_1_)	Total Passenger Travel Time (Z_2_)	Total Time (Z= 2 × Z_1_ + Z_2_)
1st form	5 local trains	1st	Local train	490.3	2.1	178.7	86.33%	1598	138,833	710,145
		2nd	Local train	490.3	2.1	178.7	86.33%	1598	138,833	
		3rd	Local train	490.3	2.1	178.7	86.33%	1598	138,833	
		4th	Local train	490.3	2.1	178.7	86.33%	1598	138,833	
		5th	Local train	490.3	2.1	178.7	86.33%	1598	138,833	
2nd form	3 local trains and 2 rapid trains	1st	Local train	490.3	2.1	178.7	86.33%	1598	138,833	698,179
		2nd	Rapid train	561.2	4.1	166.4	75.62%	3260	129,255	
		3rd	Local train	458.7	2.2	178.9	97.04%	1674	138,952	
		4th	Rapid train	561.2	4.1	166.4	75.62%	3260	129,255	
		5th	Local train	458.7	2.2	178.9	97.04%	1674	138,952	
3rd form	3 local trains and 2 express trains	1st	Local train	490.3	2.1	178.7	86.33%	1598	138,833	690,433
		2nd	Express train	617.1	4.7	159.0	63.23%	3667	123,579	
		3rd	Local train	454.4	2.3	179.8	109.43%	1790	139,707	
		4th	Express train	617.1	4.7	159.0	63.23%	3667	123,579	
		5th	Local train	454.4	2.3	179.8	109.43%	1790	139,707	
4th form	3 local trains,1 rapid train and 1 express train	1st	Local train	490.3	2.1	178.7	86.33%	1598	138833	694,307
		2nd	Rapid train	561.2	4.1	166.4	75.62%	3260	129255	
		3rd	Local train	458.7	2.2	178.9	97.04%	1674	138952	
		4th	Express train	617.1	4.7	159.0	63.23%	3667	123579	
		5th	Local train	454.4	2.3	179.8	109.43%	1790	139707	
5th form	5 trains based on the stochastic model	1st	1101011	515.6	2.8	171.1	79.89%	2183	132,920	686,994
		2nd	1011101	483.0	2.6	171.8	75.99%	2043	133,481	
		3rd	1101011	495.7	2.8	171.1	101.57%	2183	132,920	
		4th	1011101	483.0	2.6	171.8	75.99%	2043	133,481	
		5th	1101011	495.7	2.8	171.1	101.57%	2183	132,920	

**Table 5:** Stochastic stop-skipping scheduling plan (‘0’ donates that the station is skipped; ”1”, otherwise).

Run	1	2	3	4	5	6	7
1st	1	1	0	1	0	1	1
2nd	1	0	1	1	1	0	1
3rd	1	1	0	1	0	1	1
4th	1	0	1	1	1	0	1
5th	1	1	0	1	0	1	1

**Table 6:** Station operating states.

Station Stop	1	2	3	4	5	6	7
(**a**) Two stations are skipped							
*Y_ij_*	1	0	0	1	0	0	1
(**b**) Three stations are skipped							
*Y_ij_*	1	0	0	0	1	0	1

**Table 7:** Results of performance analysis of different demands (unit: min).

Forms’ Categories	Waiting Time Per Passenger	Travel Time Per Passenger	TotalPassenger Waiting Time (Z_1_)	Total Passenger Travel Time (Z_2_)	Z= 2 × Z_1_ + Z_2_
(**a**) Results of Example 2					
1st form	2.0	131.0	9740	638,150	657,630
2nd form	3.1	128.2	14,814	624,344	653,972
3rd form	3.4	126.7	17,034	617,048	651,116
4th form	3.2	127.5	15,924	620,696	652,544
5th form	2.7	128.4	12,896	625,301	651,093
(**b**) Results of Example 3					
1st form	2.0	152.9	8020	613,240	629,280
2nd form	2.9	150.1	11,808	602,092	625,708
3rd form	3.4	148.9	13,504	597,012	624,020
4th form	3.2	149.5	12,656	599,442	624,754
5th form	2.4	150.8	9740	604,707	624,187
(**c**) Results of Example 4					
1st form	2.0	144.6	10,770	778,705	800,245
2nd form	2.9	141.8	15,628	763,693	794,949
3rd form	3.3	140.6	17,592	757,091	792,275
4th form	3.1	141.2	16,610	760,392	793,612
5th form	2.6	141.8	14252	763,472	791,976
(**d**) Results of Example 5					
1st form	2.2	137.4	9730	668,400	687,860
2nd form	3.2	134.7	14,610	655,488	684,708
3rd form	3.6	133.7	16,610	650,326	683,546
4th form	3.4	134.2	15,610	652,907	684,127
5th form	2.9	135.3	12,574	658,456	683,604

## Appendix

**Table A1:** Main process of tabu algorithm.

Algorithm: Main	
**1**	Read the data and input parameters
**2**	num = 1;
**3**	bnum = 1;
**4**	listlength = 12;
**5**	list = round(rand(1,19));
**6**	**for** i = 1:19
**7**	list(i) = 0;
**8**	**end**
**9**	fxbest(bnum) = c;
**10**	fxlbest(num) = c;
**11**	**for** g = 1:300
**12**	k = 1;
**13**	**while**(k)
**14**	x0 = round(rand(19,1));
**15**	x0(1) = 1; x0(19) = 1;
**16**	**for** d = 1:18
**17**	**if** x0(d) + x0(d + 1) < 1
**18**	x0(d) = 1;
**19**	**end**
**20**	**end**
**21**	Z = skipM(x0);
**22**	**if** Z < c
**23**	k = 0;
**24**	**end**
**25**	**end**
**26**	**if** Z < fxbest(num)
**27**	fxbest(num) = Z;
**28**	Xbest = x0;
**29**	**end**
**30**	**end**
**31**	**if** fxbest(num) < fxlbest(bnum)
**32**	fxlbest(bnum) = fxbest(num);
**33**	**end**
**34**	x1 = x0;
**35**	**while**(num <= 300)
**36**	[x,p1,p2] = near(x1,list);
**37**	list = newlist(p1,p2,list);
**38**	x1 = x;
**39**	num = num + 1;
**40**	Z = skipM(x1);
**41**	fxbest(num) = Z;
**42**	**if** fxbest(num) < fxlbest(bnum)
**43**	bnum = bnum + 1;
**44**	fxlbest(bnum) = fxbest(num);
**45**	Xbest = x1;
**46**	list(p1) = 0;
**47**	list(p2) = 0;
**48**	**end**
**49**	**end**

**Table A2:** Neighborhood search of tabu algorithm.

Function 1: Near	
**1**	**function** [x,p1,p2] = near(x1,list)
**2**	temp = c; xtemp = round(rand(19,1)); p1 = 1; p2 = 1;
**3**	**for** j = 1:100
**4**	k = 1;
**5**	xt = x1;
**6**	**while**(k)
**7**	px = ceil(rand*18)+1;
**8**	py = ceil(rand*18)+1;
**9**	xt(px) = abs(xt(px) − 1);
**10**	xt(py) = abs(xt(py) − 1);
**11**	**for** d=1:18
**12**	**if** xt(d) + xt(d + 1) < 1
**13**	xt(d) = 1;
**14**	**end**
**15**	**end**
**16**	xt(1) = 1;xt(19) = 1;
**17**	Z = skipM(xt);
**18**	**if** Z < c
**19**	k = 0;
**20**	**end**
**21**	**end**
**22**	**if** (list(px) > 0)&&(list(py) > 0)
**23**	**continue**;
**24**	**else**
**25**	**if** skipM(xt) < temp
**26**	temp = skipM(xt);
**27**	xtemp = xt;
**28**	p1 = px; p2 = py;
**29**	**end**
**30**	**end**
**31**	**end**
**32**	X = xtemp;
**33**	p1 = p1’;
**34**	p2 = p2’;

**Table A3:** List update of tabu algorithm.

Function 2: Newlist	
**1**	**function** list = newlist(pxj,pyj,list)
**2**	**for** m = 1:19
**3**	**if** list(m) > 0
**4**	list(m) = list(m) − 1;
**5**	**end**
**6**	**end**
**7**	list(pxj) = 10;
**8**	list(pyj) = 10;
**9**	list = list’;

**Table A4:** Skip rule of tabu algorithm.

Function 3: SkipM	
**1**	**function** Z = skipM(x)
**2**	**for** i = 1:L
**3**	**for** j = 1:L
**4**	R = 0;
**5**	**for** k = (i + 1):j
**6**	R = R + r(k);
**7**	**end**
**8**	T1 = 0;
**9**	**for** k = (i + 1):(j − 1)
**10**	T1 = T1 + T × x(k);
**11**	**end**
**12**	Z1 = Z1 + Q5(i,j) × (h/2 × x(i) × (0.5 + 0.5 × x(j)) + (1 − x(i) × (0.5 + 0.5 × x(j))) × 3 × h/2);
**13**	Z2 = Z2 + Q5(i,j) × (R + T1);
**14**	**end**
**15**	**end**
**17**	Z = Z1 + Z2;
**18**	**for** i = 1:L
**19**	Z3 = Z3 + r(i) + T × x(i);
**20**	**end**
**21**	Z3 = Z3−2 × T;
**22**	Z = Z + Z3;
